# A new method of adhesive system application improves the bond strength between fiber post and root dentin

**DOI:** 10.1590/0103-6440202405720

**Published:** 2024-04-10

**Authors:** Rafael Nesello, Leonardo Thomasi Jahnke, Wesley Misael Krabbe, Charles André Dall Agnol, Manuela Favarin Santini, Lucas Machado Silveira, Leonardo Miotti, Marcus Vinícius Reis Só, Ricardo Abreu da Rosa

**Affiliations:** 1 Department of Conservative Dentistry, School of Dentistry, Federal University of Rio Grande do Sul, Porto Alegre, RS, Brazil.; 2 Department of Stomatology, Federal University of Santa Maria, Santa Maria, RS, Brazil

**Keywords:** glass fiber posts, adhesive systems, rotary brush, ultrasonic activation

## Abstract

This study evaluated a new method of adhesive system application on the bond strength between fiber post and root dentin using two adhesive systems. The canals of sixty bovine incisors were prepared and obturated. The roots were divided into six groups (n=10) according to the adhesive system (Clearfil SE - CSE and Single Bond Universal - SBU) and the application strategy (microbrush - MB; rotary brush - RB; and ultrasonic tip - US). The glass fiber posts were cemented with resin cement (RelyX ARC). The roots were sectioned perpendicularly to their long axis, and three slices per root were obtained. Previously to the push-out test, confocal laser scanning microscopy (CLSM) was performed to illustrate the interfacial adaptation of the cement to the root canal walls. Failure patterns were analyzed with 40x magnification. Shapiro-Wilk indicated a normal distribution of the data. The bond strength values were compared using one-way ANOVA and Tukey's tests. Student's T test analyzed the differences between the adhesive systems within each third and protocol. A significance level of 5% was used. CSE with RB showed higher mean bond strength values compared to MB (conventional technique) (P < 0.05). US application resulted in intermediate bond strength values for CSE (P > 0.05). The application of SBU using RB generated higher mean bond strength values compared to MB and US (P < 0.05). Adhesive failures were predominant (65.5%). CSE and SBU application with the new rotary brush improved the bond strength of fiber posts to root dentin compared to the conventional strategy.

## Resumo

Este estudo avaliou o efeito de um novo método para aplicação do sistema adesivo na resistência de união entre pino de fibra e dentina radicular usando dois sistemas adesivos. Os canais de sessenta incisivos bovinos foram preparados e obturados. As raízes foram divididas em seis grupos (n=10) de acordo com o sistema adesivo (Clearfil SE - CSE e Single Bond Universal - SBU) e com a estratégia de aplicação (microbrush - MB, escova rotatória - RB e inserto ultrassônico - US). Os pinos de fibra foram cimentados utilizando cimento resinoso (RelyX ARC). As raízes foram seccionadas perpendicularmente ao seu longo eixo e três fatias foram obtidas. Previamente ao teste de push-out, foi realizada microscopia de varredura confocal a laser para ilustrar a adaptação interfacial do cimento às paredes do canal radicular. Os padrões de falha foram analisados com magnificação de 40x. O teste de Shapiro-Wilk indicou distribuição normal dos dados. Os valores de resistência de união foram comparados usando o teste ANOVA de uma via e post hoc de Tukey. As diferenças entre os sistemas adesivos em cada terço e de acordo com cada protocolo foram analisadas pelo teste T de Student. O nível de significância foi de 5%. CSE aplicado com RB apresentou maiores valores médios de resistência de união do que quando aplicado com MB (técnica convencional) (P<0,05). Aplicação com US apresentou valores intermediários de resistência de união para o CSE (P>0,05). A aplicação do SBU utilizando RB gerou maiores valores médios de resistência de união comparados com MB e US (P<0,05). Falhas adesivas foram predominantes (65,5%). A aplicação do CSE e SBU com a nova escova rotatória melhorou a resistência de união dos pinos de fibra à dentina radicular comparados com a estratégia convencional.

## Introduction

Restoring endodontically treated teeth with extensive coronal destruction is a complex procedure. Among the available strategies is the restoration retained by glass fiber posts, which have mechanical properties like dentin and can dissipate forces throughout the remaining tooth structure, minimizing the chances of long-term root fracture ^1^.

Glass fiber posts are associated with the use of resin cement agents. Resin cements have emerged in the market to address the shortcomings of their predecessors, offering good retention ^2^ and superior physical and mechanical properties ^3^ compared to materials like zinc phosphate cements, in addition to being more esthetic ^4^. In general, resin cements can be classified into two categories: conventional cements that require an adhesive system and self-adhesive or self-etch cements that do not require prior treatment of the tooth substrate ^5^.

Conventional resin cement should be used after treating the root dentin with adhesive systems, depending on the adhesive strategy adopted. Self-etch adhesives, commonly used with dual-cure resin cement, are available either as a single-bottle system containing primer and adhesive (referred to as universal adhesives) or as a two-bottle system with separate primer and adhesive components ^6^. This class of adhesives allows for greater technical versatility since they can promote bonding to dentin with or without prior phosphoric acid etching ^6^. Using this class of adhesives can be advantageous in root canals, where access to root dentin is limited ^6^.

Clinically, accessing the root dentin substrate is challenging due to the difficulty of access. In this regard, application methods of adhesive systems can positively contribute to adhesive cementation procedures ^7^. Ultrasonic (US) activation is used in various endodontic procedures, including the activation of irrigation solutions ^8^, allowing them to penetrate anatomically complex areas and dentinal tubules, resulting in enhanced cleaning ability of the root canal system ^9^. A recent study even recommends US activation of intracanal medication and endodontic cement before the root canal filling to increase the ion dissociation of calcium hydroxide and reduce the number of areas unfilled by the obturator cement ^10^. Some studies have investigated the sonic and US application of adhesive systems to improve the adhesion of fiber posts cemented inside root canals ^11^ ^,^ ^12^.

**Figure 1:**
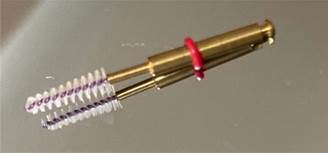
Rotary brush (MK Life)

In this context, a new technique of adhesive system application is proposed, which involves using an intracanal brush attached to a low-speed contra-angle handpiece to mechanically activate the adhesive against the walls of the root canal (Figure 1). This rotary brush (RB) (MK Life, Porto Alegre, RS, Brazil) was introduced to the market in 2021 to effectively remove debris from the post space after preparation according to the manufacturer. The possible use of this RB to apply adhesive systems inside the root canal is due to its low cost and ease of use compared to other techniques. Furthermore, this idea is based on a previous study that found benefits in the active application of adhesive systems on root canal walls ^13^.

Therefore, this study aimed to evaluate the effect of a new method of adhesive system application on the fiber post-bond strength to root dentin. The null hypotheses are that there will be no difference in bond strength values among the application methods, between the two adhesive systems (two-step self-etch and one-step self-etch), and among different regions of the post space (cervical, apical, and middle) within the same experimental group.

## Materials and methods

### Ethical Considerations

The Research Committee of the School of Dentistry approved the research project, Federal University of Rio Grande do Sul.

### Sample Acquisition

The following parameters were considered for the sample calculation, as indicated by Rosa et al. ^13^: the minimum difference between treatment means = 1.65, standard deviation of the error = 0.92, number of treatments = 6, test power = 0.80, and significance level = 0.05. Thus, sixty bovine central incisors were used. After extraction, all teeth were stored in a 0.2% thymol solution (Spengler Farmácia de Manipulação, Porto Alegre, RS, Brazil). The periodontal tissue adhered to the root surface was removed using #15 surgical blades (Swann-Morton, Sheffield, England). All roots were examined under a 4× magnifying glass. Teeth with root curvatures greater than 20º, initial anatomical diameter larger than what is compatible with a K-file #50, root resorption, fractures, or cracks were excluded and replaced with others that met the inclusion criteria.

### Sample Preparation

The root remnants were standardized to a length of 15 mm perpendicular to the long axis of the root using a double-sided diamond disc (Komet, Santo André, SP, Brazil) in a low-speed handpiece under abundant irrigation. Canal exploration was performed using a #20 file (Dentsply Sirona, Charlotte, NC, USA) until its visualization at the apical foramen, and the working length (WL) was established as 1 mm short of the root length.

The canals were prepared using a step-back technique up to a K-type file #80 (Dentsply Sirona) under irrigation with 20 mL of 2.5% sodium hypochlorite (NaOCl) (Farmácia Marcela, Porto Alegre, RS, Brazil). At the end of the preparation, the canals were irrigated with 5 mL of 2.5% NaOCl, followed by 5 mL of saline solution, and 5 mL of 17% EDTA (Fórmula e Ação, São Paulo, SP, Brazil) for 3 minutes. A final irrigation with 10 mL of saline solution was performed to remove the EDTA. All irrigation procedures were performed using 5-mL syringes (Ultradent Products Inc., South Jordan, UT, USA) and Endo-Eze Tip needles (Ultradent), inserted 3 mm short of the apical foramen.

Before root canal filling, the canals were dried with #80 paper points (Tanariman Industrial Ltda, Manacapuru, AM, Brazil). AH Plus (Dentsply Sirona) was handled according to the manufacturer's instructions and placed in the canal using XP Clean instruments (MK Life, Porto Alegre, RS, Brazil) set at 13 mm and a speed of 800 rpm with 1 N/cm of torque. The root canal filling technique used was cold lateral compaction, with a #80 gutta-percha cone (Tanariman) and B8 accessory cones (Tanariman).

After root canal filling, the roots were restored with temporary restorative material (Cimento Obturador Provisório; Villevie, Joinvile, SC, Brazil) and kept in a humid environment using gauze-soaked in saline solution at 37°C for fifteen days to allow complete setting of the endodontic cement ^14^. Next, the root canal filling was partially removed using #3 Largo burs (Dentsply Sirona), leaving approximately 4 mm of filling material at the apical third of the root. Radiographs were taken to confirm the complete removal of the filling material from the canal walls.

Subsequently, the canals were prepared with the drill corresponding to the post size (Exacto 2, Angelus, Londrina, PR, Brazil) to a depth of 10 mm. Then, the canals were irrigated with 5 ml of 5% NaOCl followed by 5 ml of 17% EDTA for 60 seconds (s) each, using 5-ml syringes (Ultradent) and Endo-Eze Irrigator Tip needles (Ultradent). Finally, the canals were dried with #80 absorbent paper points (Dentsply Sirona).

The adaptation of the fiber posts in each canal was checked. Then the posts were cleaned with 70% ethanol (Mega Química Ind. Comércio Ltda, Pederneiras, SP, Brazil), and a silane bonding agent (Angelus, Londrina, PR, Brazil) was applied for 1 minute.

### Experimental Groups

The roots were randomly divided (http://www.random.org/integers/) into six groups (n=10) according to the adhesive system and the application strategy of the adhesive used:

Two adhesive systems were evaluated: one-step self-etch (Single Bond Universal - 3M ESPE, St. Paul, MN, USA) and two-step self-etch (Clearfil SE - Kuraray, OK, Japan). They were applied using three strategies (microbrush - MB; rotary brush - RB, and ultrasonic tip -US). Box 1 presents the experimental groups and describes the protocols for applying the adhesive systems before the cementation of the fiber posts.

**Box 1:**
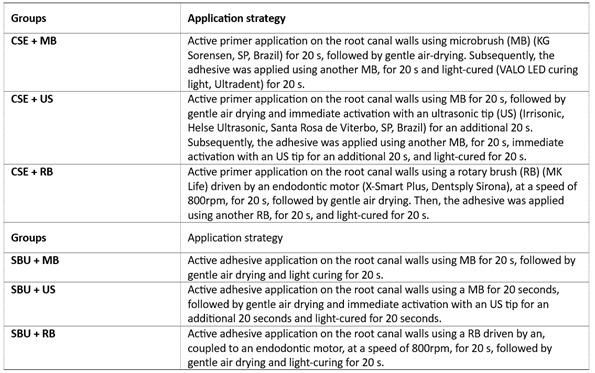
Design of the experimental groups in the study according to the adhesive system application strategy.

To each adhesive system applied on the root surface, powder of Rhodamine B (Sigma-Aldrich, Chemie GmbH) was incorporated at a rate of 1% of the adhesive weight to allow for subsequent visualization under confocal laser scanning microscopy (CLSM). The adhesive weight was measured on a precision digital scale (Mettler-Toledo Ind. e Com. Ltda., Barueri, SP, Brazil). The RelyX ARC dual-cure resin cement (3M ESPE, St. Paul, MN, USA) was manipulated according to the manufacturer's recommendations and carried into the canal using a #2 Lentulo spiral (Dentsply Sirona). The post was positioned, and excess cement was removed with a microbrush. A previously calibrated light-emitting diode (LED) polymerization unit (Radii Cal; SDI) light-cured the resin cement for the 40s.

The specimens were stored at 37°C for 24 hours. Afterward, the roots were sectioned perpendicularly to their long axis in a cutting machine (LabCut 1010; Extec Corp, CT, USA) using a diamond disc (ERIOS Equipamentos, São Paulo, SP, Brazil) operating at 280-300 rpm under continuous water refrigeration. Then, three slices with a thickness of 2.0 mm ± 0.3 mm were obtained, one for each region of the post (cervical, middle, and apical). They were then polished with decreasing grit sandpaper strips up to 1200 and discs with polishing paste (Arotec, Cotia, SP, Brazil), followed by rinsing with distilled water to remove any polishing residue. A single operator performed all experimental procedures.

Interfacial adaptation to root dentin and intratubular penetration using confocal laser scanning microscopy

Descriptive images using confocal laser scanning microscopy (CLSM) (Olympus Corporation) were performed to illustrate the interfacial adaptation of the cement to the root canal walls and the presence of voids or empty spaces. The absorption and emission wavelengths for rhodamine B were 540 nm and 494 nm, respectively. Additionally, stereo microscopy images were captured at a magnification of 10×.

### Bond strength test

For the bond strength test, each slice was positioned in a metallic device with a central opening (diameter of 3 mm) larger than the canal's diameter. The coronal side of the slice was placed in contact with the metallic device. Then, a cylindrical metal tip (diameter of 0.8 mm) applied a load on the fiber post until failure. The push-out bond strength test was performed on a universal testing machine (EMIC, São José dos Pinhais, SP, Brazil) at a 0.5 mm/min crosshead speed. The bond strength values were obtained in megapascals (MPa) using the formula described in Valandro et al. ^15^. All measurements were obtained using a digital caliper.

Each slice was analyzed using an optical microscope (Olympus, BX60M, Tokyo, Japan) at 40× magnification by two researchers (R.N, C.D.J) to classify failure patterns. A third researcher (R.A.R.) performed the slice analysis in case of disagreement. The failure patterns were classified as follows: predominantly adhesive cement/dentin - if the cement detached from the dentin; predominantly adhesive cement/post - if the cement detached from the post; dentin cohesive - if the failure occurred within the dentin; cement cohesive - if the failure occurred within the cement; and post cohesive - if the failure occurred within the post ^16^.

### Statistical Analysis

The collected data were compiled in a spreadsheet (Microsoft Office Excel 2007, Microsoft Corporation, Redmont, WA, USA) and statistically analyzed using the SPSS for Windows software (SPSS Inc., Chicago, IL, USA). Shapiro-Wilk indicated a normal distribution of the data. Therefore, the bond strength values between the tested protocols and among the root canal thirds within the same experimental protocol were compared using one-way ANOVA and Tukey's post hoc test. The difference between the adhesive systems within each third and protocol was analyzed using Student's t-test. A significance level of 5% was established.

## Results

**Figure 2:**
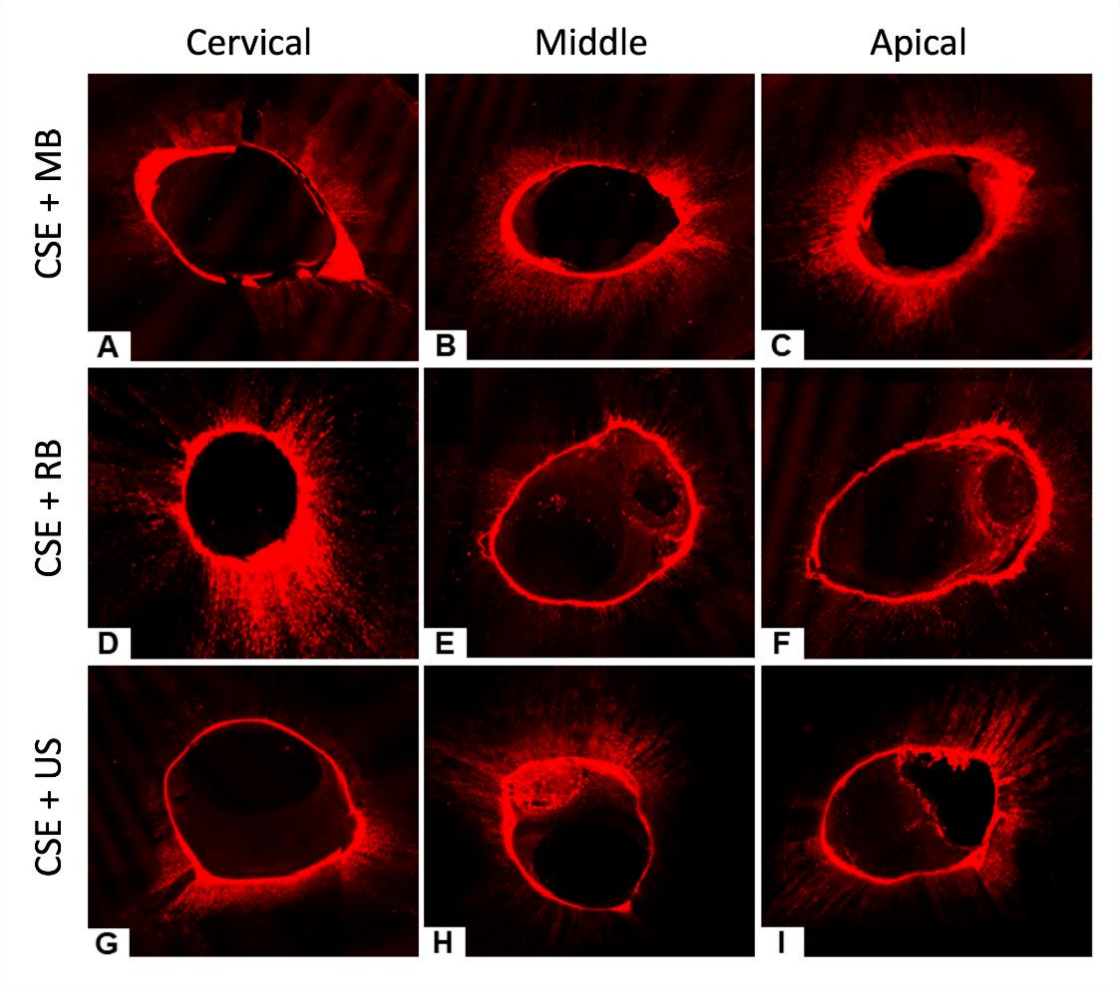
CLSM images showing the intratubular penetration of the Clearfil SE (CSE). Clearfil SE + microbrush cervical (A); middle (B); and apical third (C). Clearfil SE + rotary brush cervical (D); middle (E); and apical third (F). Clearfil SE + ultrasonic tip cervical (G); middle (H); and apical third (I).

**Figure 3:**
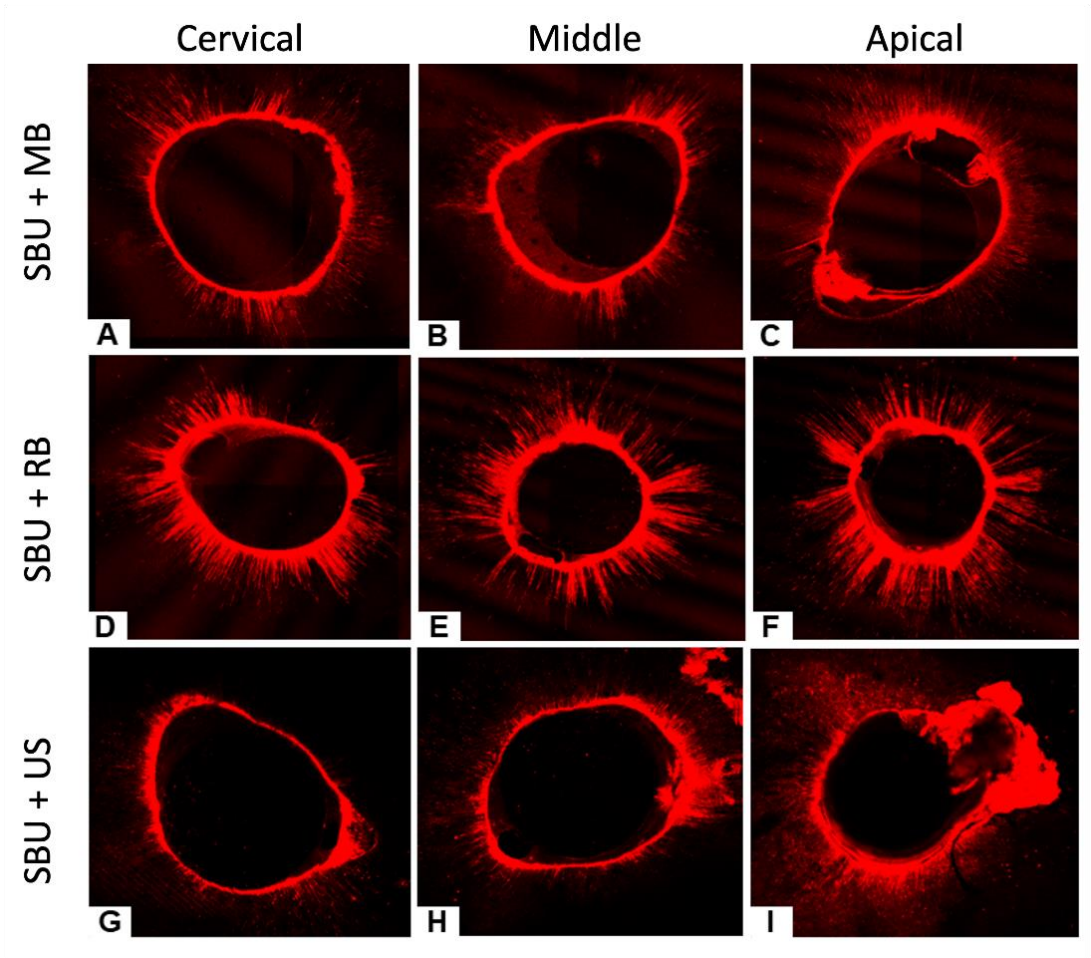
CLSM images showing the intratubular penetration of the Single Bond Universal (SBU). Single Bond Universal + microbrush cervical (A); middle (B); and apical third (C). Single Bond Universal + rotary brush cervical (D); middle (E); and apical third (F). Single Bond Universal + ultrasonic tip cervical (G); middle (H); and apical third (I).

Figures 2 and 3 show the adhesive system penetration obtained through CLSM for the groups where CSE and SBU adhesive systems were used, respectively.

In the intergroup analysis, the application protocol of Clearfil SE Bond (CSE) (Kuraray, OK, Japan) adhesive system using RB showed higher mean bond strength values compared to the microbrush (MB) application (conventional technique) (P < 0.05). US application resulted in intermediate bond strength values for CSE (P > 0.05). The application of SBU (3M ESPE) using RB generated higher mean bond strength values compared to the conventional technique (MB) and US (P < 0.05). Table 1 presents the bond strength values according to the protocols for applying adhesive systems in the root dentin.

**Table 1:** Bond strength values for the experimental groups after the push-out test (mean ± standard deviation)

	Microbrush (MB)	Ultrasonic tip (US)	Rotary brush (RB)
Clearfil SE (CSE)			
Apical	3.69 + 2.04 Bb	5.58 + 2.24 ABb	8.78 + 3.96 Ab
Middle	5.42 + 3.71 Aab	7.37 + 3.20 Aab	8.27 + 4.94 Ab
Cervical	10.16 + 7.75 Aa	9.57 + 2.22 Aa	12.17 + 4.05 Aa
Mean	6.42 + 3.48 B	7.09 + 1.99 AB	9.95 + 3.55 A
Single Bond Universal (SBU)			
Apical	4.72 + 3.26 Ba	4.58 + 4.29 Ba	9.68 + 5.05 Aa
Middle	5.60 + 2.96 Ba	7.10 + 3.53 Ba	12.57 + 5.28 Aa*
Cervical	7.56 + 2.43 Ba	6.36 + 2.46 Ba*	12.80 + 3.63 Aa
Mean	6.32 + 1.73 B	5.91 + 2.61 B	11.63 + 3.99 A

Uppercase letters compare the application protocols within each region of the post after one-way ANOVA and Tukey's post hoc test (P < 0.05). Lowercase letters compare the regions of the post within each adhesive system application protocol after one-way ANOVA and Tukey's post hoc test (P < 0.05). * indicates a difference between adhesive systems combining the application technique and the portion of the post after the Student's t-test (P < 0.05).

In the apical portion of the post, higher bond strength values were found when CSE was applied using RB compared to MB application (conventional technique) (P < 0.05). In the cervical and middle portions of the post, the bond strength values were similar, regardless of the application protocol (P > 0.05). For the SBU adhesive system, the application with RB resulted in higher bond strength values compared to the MB (conventional technique) and US, regardless of the post region (P < 0.05). The bond strength values of the two tested adhesive systems were similar (P > 0.05). Except for the cervical portion of the post when the US application was performed (P < 0.05) and in the middle portion of the post when the adhesive systems were applied with RB (P < 0.05).

Table 2 presents the failure patterns that occurred after the bond strength test. There were many adhesive failures (65.5%), with 49.4% predominantly adhesive at the cement/dentin interface and 16.1% predominantly adhesive at the cement/post interface. Cohesive dentin failures were observed in 28.9% of the samples, especially in the groups where SBU was used.

**Table 2:** Distribution of failure patterns for the experimental groups after the bond strength test.

Strategy	ACD	ACP	CD	CC	CP
CSE + MB	20	7	2	-	1
CSE + US	14	3	10	-	3
CSE + RB	13	5	9	-	3
SBU + MB	20	2	7	-	1
SBU + US	14	2	12	-	2
SBU + RB	8	10	12	-	-
Total	89	29	52	0	10
	(49.4%)	(16.1%)	(28.9%)	(0%)	(5.6%)

ACD = Predominantly Adhesive Cement/Dentin Interface; ACP = Predominantly Adhesive Cement/Post Interface; CD = Dentin Cohesive; CC = Cement Cohesive; CP = Post Cohesive; D = Discarded. CSE = Clearfil SE; SBU = Single Bond Universal; MB - microbrush; US = ultrasonic tip; RB = rotary brush.

## Discussion

This study evaluated the bond strength between fiber posts and root dentin using two self-etch adhesive systems applied with a new rotary brush method. The null hypotheses were rejected, as there were differences between the adhesive systems and the protocols used.

CLSM was used to indicate the penetration of the adhesive system into the dentinal tubules according to the protocols tested. Two samples per group were polished with sandpaper strips and polishing paste to improve the visualization in the CLSM. The confocal laser-scanning microscope operated at a wavelength of 540 nm and 494 nm for rhodamine B dye. Therefore, a control (without rhodamine) was not used. The laser will indicate only the regions where the dye reaches with the adhesives. The CLSM images in the SBU groups showed greater penetration when activated with RB, with no significant differences between MB and US. In CSE groups, greater penetration was observed in the cervical third when the adhesive was activated with RB and in the apical third when activated with the MB. The present study's findings reveal that the use of RB proved to be effective in the activation process of the adhesive systems. Verdum et al. ^12^, using CLSM images, demonstrated that the CSE activated by ultrasound showed a high penetration into the dental substrate ^12^. Although the US presents a uniform pattern of adhesive penetration, especially in the middle and apical thirds, RB promotes greater penetration in certain canal regions.

The apical thirds presented lower bond strength values than the other two-thirds of post space, regardless of the application protocols and adhesive system used. Possible reasons for this include potential porosities in the hybrid layer ^15^ and difficulties in complete polymerization of the materials involved in establishing adhesion. These phenomena are related to the anatomical complexity of the apical region, where there is limited access to the polymerization-activating light source and the materials used for adhesive and resin cement application. Consequently, empty spaces may occur in the adhesive interface, hindering proper interaction between the adhesive and the dental substrate. Inadequate interaction in this interface can result in air entrapment in the most apical region of the post space, creating possible failures at this interface ^17^. This influence of the anatomical region of the canal is evident in the performance of both adhesive systems in the cervical region, where they exhibited better adhesive performance than the apical third.

Regarding the cleaning of the prosthetic canal, studies have shown the influence of reducing remnants of obturating materials from the canal walls ^18^, as endodontic sealer and gutta-percha can obstruct dentinal tubules, affecting dentin permeability and reactivity, and thus impacting the adhesive interface and bond strength ^19^ ^,^ ^20^. Kul et al. ^21^ observed the highest bond strength values when the prosthetic canal was irrigated with 5 mL of 5% NaOCl, followed by 5 mL of 17% EDTA. The same cleaning protocol described by Kul et al. ^21^ was employed in the present study. This protocol allowed high bond strength values to be observed in some tested groups, mainly when SBU was applied using the RB. The cleaning protocol can provide favorable conditions for adequate adhesive performance of the conventional resin cement (RelyX ARC) associated with adhesive systems.

The adhesives CSE and SBU contain functional monomers with an acidic composition. These monomers can incorporate the smear layer, making it part of the hybrid layer ^15^. The active application of adhesive systems has been proven to promote higher bond strength values in dentin ^13^. Self-etch adhesive systems can further benefit from the positive effect of an active application, as this protocol can promote greater resin tag penetration and, consequently, improve the bond durability of this category of adhesives ^22^. US application also considered an active adhesive application method, has been shown in previous studies to promote increased penetration of self-etch adhesives into dentinal tubules, resulting in improved bond strength values ^12^ ^,^ ^23^. In this study, the CSE adhesive performed better when subjected to both tested methods of active application compared to conventional application, with equivalent performance between RB and US. It showed the best results when applied with RB, regardless of the post portion. In the present study, the one-step adhesive did not benefit from US application, only RB. The viscosity of the two adhesive systems may be the main reason to explain this difference in their behavior. The higher viscosity of SBU may negatively interfere with the positive effect of US, making this method insufficient to promote closer contact of the adhesive with the substrate. The intense friction promoted by the RB can apply the adhesive more vigorously, which is compatible with enhancing the performance of this adhesive in root canals.

One aspect associated with adhesion to root dentin that deserves attention is the limited ability of conventional resin cement to dissipate stresses generated during polymerization shrinkage ^24^. The cavity configuration factor (C-Factor) is related to the ratio between the bonded and accessible areas, which is significantly elevated inside the root canal. The polymerization shrinkage can cause gaps in the adhesive interface between the cement and the post and between the cement and the dentin, affecting the bond strength and leading to such failures after the push-out test ^25^. In this study, as observed in previous studies ^11^ ^,^ ^14^ ^,^ ^17^, adhesive failures (65.5%) were predominance after the push-out test. Among these, 49.4% were cement/dentin adhesive failures, and 16.1% were cement/post-adhesive failures. The high C-Factor observed inside the root canal, the difficulty in controlling dentin moisture, and the challenge of photopolymerizing the adhesive system and resin cement inside the root canal, especially in deeper areas, contribute to these failures ^25^. Cohesive dentin failures, where specimen fracture occurs, were observed in 28.9% of the samples, especially in the groups where adhesive systems were applied with RB or US.

The resin cement and adhesive system directly relate to two substrates: root dentin and the fiberglass post. This complex adhesive chain, where each material and substrate can be considered a link, relies on the correct bonding between them. A failure in any interface can compromise the canal's post-retention and the restoration's clinical longevity ^17^ ^,^ ^18^ ^,^ ^19^ ^,^ ^20^ ^,^ ^21^. In this context, cohesive failures were excluded from the bond strength calculation since they do not effectively represent the bonding strength between the materials involved in post-cementation to dentin ^26^. The push-out test was used in this study because it can create a uniform stress concentration area at the adhesive interface, favoring the occurrence of such failures ^27^. However, it is essential to note that this laboratory test conducted under controlled conditions should not be solely relied upon to indicate or contraindicate clinical decisions ^18^. Further studies investigating the effects of adhesive system application methods are still needed to endorse the effectiveness of the tested protocols.

The present study aimed to evaluate different methods of adhesive system application during the resin cementation process of fiberglass posts. For this purpose, a conventional dual resin cement was used, which requires a prior application of an adhesive system. The RelyX ARC cement (3M ESPE, St. Paul, MN, USA), used in all experimental groups, was chosen because it is widely used clinically and has ample evidence of adhesive performance, often as a control in studies. Additionally, the influence of the resin cement factor on the adhesive process was not investigated. Thus, using cement without self-etching functional monomers was necessary to isolate this variable. There are reports in the literature of possible incompatibility between acidic monomers and dual resin cement, as these monomers can interfere with the polymerization of resin cement containing camphorquinone and tertiary amine in their composition, thereby compromising the degree of conversion ^28^. This negative effect is minimized when the functional monomers can adequately interact with the calcium derived from hydroxyapatite in the dental structure, leading to acid-base neutralization and consequently avoiding the inactivation of the tertiary amine co-initiator, which activates the chemical pathway of polymerization ^28^ ^,^ ^29^. Based on the results of the present study, we can speculate that the application of the adhesive inside the canal promotes this interaction between monomers and hydroxyapatite, as the groups that underwent US application or RB showed higher bond strength values compared to the control. The active application of the adhesive systems used in this study, which contain 10-MDP in their composition, prevented possible incompatibility and impairment in the polymerization of the resin cement. Further studies are needed to elucidate the mechanism of RB in the adhesive application process and its behavior with different types of adhesive systems and other resin cementation systems, including universal cement (with functional monomers in their composition).

Based on the present study's results, applying self-etch adhesive systems in the root canal using RB proved to be a viable, effective, and low-cost method. The application with RB generated higher bond strength values than the conventional application method for both SBU and CSE. The RB was also superior to the US for the application of the SBU adhesive system.
